# Assessment of Potential Herb-Drug Interactions among Nigerian Adults with Type-2 Diabetes

**DOI:** 10.3389/fphar.2016.00248

**Published:** 2016-08-10

**Authors:** Udoamaka Ezuruike, Jose M. Prieto

**Affiliations:** Department of Pharmaceutical and Biological Chemistry, Centre for Pharmacognosy and Phytotherapy, University College London School of PharmacyLondon, UK

**Keywords:** herb-drug interactions, diabetes, Nigeria

## Abstract

It is becoming increasingly evident that patients with diabetes do not rely only on prescription drugs for their disease management. The use of herbal medicines is one of the self-management practices adopted by these patients, often without the knowledge of their healthcare practitioners. This study assessed the potential for pharmacokinetic herb-drug interactions (HDIs) amongst Nigerian adult diabetic patients. This was done through a literature analysis of the pharmacokinetic profile of their herbal medicines and prescription drugs, based on information obtained from 112 patients with type-2 diabetes attending two secondary health care facilities in Nigeria. Fifty percent of the informants used herbal medicines alongside their prescription drugs. Worryingly, 60% of the patients taking herbal medicines did not know their identity, thus increasing the risk of unidentified HDIs. By comparing the pharmacokinetic profile of eight identified herbs taken by the patients for the management of diabetes against those of the prescription drugs, several scenarios of potential HDIs were identified and their clinical relevance is discussed. The lack of clinical predictors points toward cultural factors as the influence for herb use, making it more difficult to identify these patients and in turn monitor potential HDIs. In identifying these possible interactions, we have highlighted the need for healthcare professionals to promote a proactive monitoring of patients' use of herbal medicines.

## Introduction

Herbal medicines are becoming an increasing component of Western pharmacotherapy mostly due to the perception that “natural” means “safe” (Brantley et al., 2014). As a result, a large number of patients with diabetes use them alongside their prescription drugs in disease management (Argáez-López et al., 2003; Kumar et al., 2006; Hasan et al., 2009); with evidence suggesting a higher use amongst those with more severe diabetic conditions (Nahin et al., 2012). One reason for this might be because of the difficulty in attaining adequate glycaemic control in type-2 diabetic patients despite existing pharmacotherapy, due to the steady decline in beta cell function (Robertson et al., 2004). Poor glycaemic control increases the risk of diabetic related complications ensuing in more complex treatment plans which the patients have to deal with for a long time. This characteristic of the disease lends itself as a condition whose patients are likely to seek out alternative forms of treatment (Chang et al., 2007). Unfortunately, this high prevalence is accompanied by poor communication between patients and clinicians. In the US for instance, patients with diabetes were identified as the least likely to inform healthcare practitioners of their use of herbal medicines (Saydah and Eberhardt, 2006).

With the use of herbal medicine becoming more widespread, a lot of focus has been given to evaluating them for possible herb-drug interactions (HDIs), particularly pharmacokinetic interactions since pharmacodynamic interactions are often easier to identify and monitor. Efforts have also been made to collate existing information about herbal medicines as a guide to healthcare practitioners, and as a means of forestalling potentially clinically relevant HDIs (Ulbricht et al., 2008; Williamson et al., 2013). These resources are however lacking in information about medicinal plants used in African countries, despite its proportionately high frequency of use.

The implications of clinically relevant HDIs can have far-reaching effects for most African populations, such as Nigeria, with an inadequate healthcare system (Anyika, 2014). By anticipating potential toxicities or possible herb-drug interactions, clinically relevant HDIs with significant risks can be avoided. To address this issue, a fieldwork study to identify the herbal medicines used by diabetic patients in Nigeria in their disease management was carried out. The objective of the study was to assess the potential for pharmacokinetic HDIs amongst this “at risk” group based on available information about herbal medicines and the pharmacokinetic profile of the prescribed drugs used by the patients.

## Research design and methods

### Place of study and data collection

A fieldwork study was conducted in August and September 2012 in the Pharmacy departments of two secondary health care facilities—the General hospital in Lagos Island and Central hospital in Benin City—two urban cities in the southern part of Nigeria. The study-including the proposed questionnaire to be administered—was approved by the ethics committee of UCL (Ethics Application 4124/001); and prior consent was also obtained from the ethics committee of both hospitals.

During the study period, every prescription that was identified as having one or more oral hypoglycaemic drugs during the “prescription assessment stage” was assigned to the interviewer for drug dispensing and counseling and possible inclusion of the patient in the study. Each patient was thereafter given a brief description of the study. However, only patients who gave their verbal consent to be asked additional questions relating to their use of herbal medicines, based on a semi-structured questionnaire were included. These questions were asked while drugs were being dispensed and medication counseling was given to the patients afterwards.

Information obtained from the questionnaire include demographic background of respondents including age and sex, information on the identity of the herbs and prescription drugs used (where available) either for diabetes or other co-morbidities as well as the monthly cost of prescription. Herbal medicines as described in this study include the following: supplements containing extracts of medicinal plants, un-standardized herbal preparations from herbalists, herbal preparations made by the patients themselves using plant parts purchased in herbal markets or collected from their own gardens.

No samples of herbal medicines were collected as part of the study. The identity of the herbal medicines used, were provided by the patients either as local names or its common name in English. Various literature sources were consulted to determine the botanical names of plants from their given local names. Verification of their local names were done by Mr. Ibrahim Muazzam, an ethnobotanist and staff of the department of medicinal plant research and traditional medicine, National Institute for Pharmaceutical Research and Development (NIPRD) and by staff of the Forestry Research Institute of Nigeria (FRIN) in Ibadan and assignment of the correct botanical names was done based on www.plantlist.org.

Assessment of the potential HDIs was done by analysing the prescription drugs and herbal medicines used by the patients against published information about any known pharmacokinetic effects to identify possible overlapping effects that might pose a risk.

### Statistical analyses

The descriptive and inferential statistics to determine measures of frequency and relationship between variables were carried out using IBM SPSS statistical package (version 22, IBM U.S.A.). Chi square test was used to test the association of herb use with patients' background characteristics, such as gender, age groups, presence of co-morbidities, and cost of monthly prescription. A significant relationship between variables was identified if *p* < 0.05.

## Results

### Patients' background characteristics and health status

A total number of 112 patients with type-2 diabetes, 37 from Central hospital Benin-city and 75 from General hospital Lagos gave their consent and were interviewed for the study. A summary of the background characteristics of the interviewed patients is given in Table 1. Five age categories were created: < 30 years, 31–45 years, 46–60 years, 61–75 years, and >75 years. Most of the patients were aged between 61 and 75 years (50.9%), followed by those between 46 and 60 years (34.8%).

**Table 1:** **Tabular summary of data from patients with type-2 diabetes**.

**Background characteristics**		**^#^Patients (%)**	**Use of herbal medicine (%)**	
			**Yes**	**No**
Gender	Male	43 (38.4)	23 (20.5)	20 (17.9)
	Female	69 (61.6)	33 (29.5)	34 (30.4)
Age groups (years)	≤ 30	1 (0.9)	0 (0.0)	1 (0.9)
	1–45	9 (8.1)	4 (3.6)	5 (4.5)
	46–60	39 (34.8)	19 (16.9)	20 (17.9)
	61–75	57 (50.9)6	30 (26.8)	25 (22.3)
	>75	(5.4)	3 (2.7)	3 (2.7)
Presence of co-morbidities	Hypertension + Arteriosclerosis + Arthritis	1 (0.9)	0 (0.0)	1 (0.9)
	Hypertension + High cholesterol + Arthritis	6 (5.4)	4 (3.6)	2 (1.8)
	Hypertension + High cholesterol + Arteriosclerosis	1 (0.9)	1 (0.9)	0 (0.0)
	Hypertension + High cholesterol	17 (15.2)	11 (9.8)	6 (5.4)
	Hypertension + Arteriosclerosis	2 (1.8)	1 (0.9)	0 (0.0)
	Hypertension + Arthritis	4 (3.6)	2 (1.8)	1 (0.9)
	Hypertension + Neuropathy	2 (1.8)	1 (0.9)	1 (0.9)
	Hypertension	46 (41.1)	22 (19.6)	24 (21.5)
	High cholesterol	1 (0.9)	1 (0.9)	0 (0.0)
	None	30 (26.8)	13 (11.6)	17 (15.2)
Monthly prescription cost (Naira)^*^	< 5000	23 (20.5)	9 (8.0)	12 (10.7)
	5001–10,000	27 (24.1)	17 (15.2)	10 (9.9)
	10,001–15,000	10 (8.9)	4 (3.6)	6 (5.4)
	>15,000	2 (1.8)	0 (0.0)	2 (1.8)

# *Information about herb use in two patients was not given*.

* *Data on cost of prescription was not obtained from all the interviewed patients (£1 ≈ 265 Naira, $1 ≈ 170 naira www.xe.com; Accessed 13th Nov 2014)*.

More than 70% of the patients had one or more chronic co-morbidity alongside their diabetes, the most common of which was hypertension. The other identified co-morbidities were arthritis, arteriosclerosis, hypercholesterolemia and neuropathy (Table 1). Fifty percent of the patients took herbal medicines alongside their prescription drugs either for diabetes, for one of the co-morbidities or for the management of diabetes-related side effects, such as erectile dysfunction. Fourteen patients had recently discontinued their use of herbal medicines. For those who gave reasons, three said it was “based on advice by their doctor,” two said it was “due to perceived side effects,” and one patient was “disappointed at its lack of efficacy.”

### Relationships between variables and predictors of herbal medicine use

Approximately 50% of all patients used herbal medicines alongside their prescription drugs, and this percentage was the same for both males and females. Almost 60% of those who used herbal medicines were older patients aged between 61 and 75 years (Table 1). This is possibly because the highest number of patients with type-2 diabetes fell within that age group. As such, there was no statistically significant association between age and herb use (*x*^2^ = 1.555, *df* = 4, *p* = 0.817).

Given that the inherent characteristics of the individual (age and sex) were not a predictor of herb use, we hypothesized that the presence of one or more chronic co-morbidity as well as a high monthly prescription cost could be an incentive for patients to use herbal medicines as evidenced by the study carried out by Nahin et al. (2012). Although there were more herb users within the group of patients with two or more chronic co-morbid conditions than the group with only one or none at all, this relationship was not statistically significant (*x*^2^ = 3.041, *df* = 3, *p* = 0.385). For the monthly cost of prescription, a higher number of patients who spend less money for their drugs (<10,000 naira) used herbs than those who had to pay more, but this relationship was also not statistically significant (*x*^2^ = 4.643, *df* = 3, *p* = 0.2). Thus, the choice of herbal medicine use was independent of the presence of co-morbidities or the monthly cost of prescriptions.

In trying to understand the divergent relationship between number of co-morbidities and monthly cost of prescription with herb use, it was observed that the prescription cost was not necessarily dependent on the number of co-morbidities the patient was being treated for, but on the medications on the patient's prescription. For instance, a patient with only diabetes may have been prescribed gliclazide by his/her doctor, which is more expensive than other types of sulphonylureas. On the other hand, another patient having diabetes alongside other co-morbidities might be prescribed a cheaper sulphonylurea, such as glibenclamide as well as other non-expensive medications for their co-morbidities resulting in lower prescription costs. Thus, a patient with more co-morbidities may not necessarily have higher prescription costs. At the same time, based on the results of the study, patients with more drugs on their prescription due to co-morbidities or those with the need to spend more money on their prescription drugs were not more likely to use herbs than those without. As a result, the presence of diabetic complications wasn't identified as a predictor for herb use among patients with type-2 diabetes.

### Pharmacological management of the patients

A summary of the drugs used in the pharmacological management of diabetes in the interviewed patients is given in Table 2. Eighty percent of the patients were prescribed more than one medication for their diabetes. This is in addition to the drugs prescribed for other existing co-morbidities. These results corroborate other studies that have shown inadequate blood glucose control with monotherapy in patients with type-2 diabetes. Thus, despite the lack of an association between the presence of co-morbidities or number of medications and the use of herbal medicines as expected, diabetic patients could still be high risk candidates for HDIs since they are likely to be on a multiple drug regimen of orally administered drugs.

**Table 2:** **Tabular summary of the pharmacological management of the patients' based on their prescribed hypoglycaemic agents**.

**Anti-diabetic drug(s)**	**No of patients**	Presence of co-morbidities^*^
Glibenclamide only	1	1
Insulin only	4	2
Insulin + Gliclazide	1	0
Metformin + Acarbose + Glibenclamide	2	1
Metformin + Acarbose + Gliclazide + Insulin	1	1
Metformin + Glibenclamide	42	25
Metformin + Gliclazide	10	8
Metformin + Gliclazide + Pioglitazone	3	3
Metformin + Glimepiride	19	16
Metformin + Glimepiride + Pioglitazone	2	2
Metformin + Insulin	3	2
Metformin + Insulin + Glibenclamide	2	1
Metformin + Pioglitazone	1	1
Metformin + Vidagliptin	1	1
Metformin only	18	17

* *The presence of co-morbidities indicates that there are other non-hypoglycaemic agents on the patient's prescription*.

Less than 10% of the patients interviewed used insulin either singly or in combination with an oral hypoglycaemic agent for their diabetes management. This isn't surprising as despite the proven efficacy of insulin in blood glucose control, its level of compliance and/or acceptance amongst patients with type-2 diabetes is still low due to its relatively higher cost compared to oral hypoglycaemic drugs and unpreferred mode of administration. Additional factors which are unique to Nigeria and other developing countries include limited availability and unreliable storage facilities, given that constant power supply isn't guaranteed. Thus, despite the decreased risk of pharmacokinetic interactions with insulin, patients are still unlikely to use it as an alternative.

### Pharmacokinetic and toxicological profile of herbal medicines used by patients

Out of the 56 patients who admitted to using herbal medicines alongside prescription drugs for their disease management, < 40% were aware of their identity. For these patients, they either grew the herbs themselves in their gardens or they purchased them from herbal markets. The remaining 60% were unaware of the identity of the herbal medicine used, primarily because these were unlabeled preparations (often multi-component) given to them by herbalists, whose constituents were not disclosed. A number of patients however said they were not very interested in finding out the identity as long as the preparation was effective. They considered herbal medicines to be relatively “safe” compared to conventional medicines. For this cohort of patients, the risk of HDIs would be greater as they wouldn't be able to provide any information to their physicians/healthcare practitioners that can enable them provide appropriate advice with regards to its efficacy and/or safety.

Twelve medicinal plants were identified among the patients who knew the composition of the herbal medicines which they used for their disease management (Table 3). An extensive review of plants used in diabetes management in Nigeria, including information about their interactions with known pharmacokinetic parameters had previously been carried out by the authors (Ezuruike and Prieto, 2014); and this was used for the literature analysis. *Camellia sinensis* was the only plant mentioned by the patient which was not in the review. An independent literature search carried out for this did not however yield any published data on studies evaluating its possible interaction with known pharmacokinetic parameters.

**Table 3:** **Tabular summary of the medicinal plants used by the patients and their pharmacokinetic profile**.

**Botanical name of medicinal plant (Common/Local name as used by patient)**	**Number of Patients (%)**	**Effect on intestinal P-gp or other absorption mechanisms**	**Effect on cytochromes or other hepatic enzymes (GSTs, ALT, AST)**	**Other toxic effects**
*Vernonia amygdalina* Delile **(Bitter leaf/Onugbu)**	13 (11.6)	Inhibits P-gp efflux activity (Oga et al., 2012)	–	–
*Ocimum gratissimum* L. **(Scent leaf/Nchonwu)**	5 (4.5)		Dose dependent increase in AST and ALT levels (Ajibade et al., 2012)	
*Musa paradisiaca* **(Plantain/Banana)**	3 (2.7)	Polyvalent cations in the plant form non-absorbable complexes with certain drugs (Nwafor et al., 2003)	–	–
*Mangifera indica* L. **(Mango)**	3 (2.7)	Inhibits the P-gp efflux activity (Chieli et al., 2009)	Inhibits CYP 1A1/2 and 3A4 activities in rat liver microsomes (Rodeiro et al., 2009)	–
*Persea americana* Mill. **(Avocado pear)**	2 (1.8)	–	Inhibits CYP 3A4/5/7 enzymes to different extents (Agbonon et al., 2010)	–
*Gongronema latifolium* Benth. **(Utazi)**	1 (0.9)	–	–	–
*Abelmoschus esculentus* (L.) Moench **(Okro)**	1 (0.9)	Water soluble fractions inhibits metformin absorption *in vivo* (Khatun et al., 2011)	–	–
*Anarcadium occidentale* L. **(Cashew)**	1 (0.9)	–	–	–
*Azadirachta indica* A. Juss. **(Neem)**	1 (0.9)	–	–	Pharmacotoxic effects of neem oil in lungs and CNS (Gandhi et al., 1988)
*Camellia sinensis* (L.) Kuntze **(Tea)**	1 (0.9)	–	–	–
*Picralima nitida* (Stapf) T. Durand and H. Durand **(Mkpokiri/Abere)**	1 (0.9)	–	Elevated AST, ALT, and GSH levels (Kouitcheu Mabeku et al., 2008)	Hepatotoxic effects (Fakeye et al., 2004)
*Terminalia catappa* L. **(Tropical almond)**	1 (0.9)	–	–	Hepatotoxic at high doses due to punicalagin (Lin et al., 2001)
**Unknown**	36 (31.9)	NA	NA	NA
**Discontinued**	14 (12.4)	NA	NA	NA

For all of the 12 identified plants, pharmacological evidence validating their traditional use in diabetes management have been published. As shown in Table 3, eight of the identified plants have also been shown to interact with known pharmacokinetic parameters, with a potential to cause HDIs when co-administered with prescription medicines. In addition, three of the plants—*Azadirachta indica* A. Juss, *Picralima nitida* (Stapf) T. Durand and H. Durand, and *Terminalia catappa* L. have been known to cause specific organ toxicities in large doses (Table 3).

### Assessment of potential herb-drug interactions

Table 4 gives a summary of the pharmacokinetic effects of the different drugs prescribed to the interviewed patients. In particular, the table highlights overlapping effects with some of the identified herbal medicines on enzymes and/or proteins that are common targets for drug interactions. This assessment shows that based on available information, all of the prescribed drugs may interact with one or more of 7 out of the 12 identified herbs (Table 4).

**Table 4:** **Map of the potential pharmacokinetic interaction between the prescribed drugs and the herbal medicines used by the patients**.

**Prescription drug**	**Pharmacokinetic effects (Sweetman, 2011; Ezuruike and Prieto, 2014)**										
	**Intestinal absorption**	**Efflux**	**Cytochromes**								**Liver toxicity**
		**P-gp**	**1A2**	**2B6**	**2C8**	**2C9**	**2C19**	**2D6**	**2E1**	**3A4**	**AST/ALT**
Glibenclamide		^a^, ^b^ VA, MI				^a^				^a^ MI, PA	
Metformin	AE	B									
Pioglitazone										^a^ MI, PA	^d^ OG, PN, TC
Sitagliptin		^a^ VA, MI									
Vidagliptin		^a^ VA									
Amlodipine		^b^, ^c^									
Indapamide										^a^ MI, PA	
Losartan						^a^				^a^ MI, PA	
Nifedipine			^b^ MI						^b^	^a^ MI, PA	
Atorvastatin		^a^, ^b^ VA, MI								^a^ MI, PA	^d^ OG, PN,TC
Clopidogrel				^a^	^a^		^a^			^a^ MI, PA	
Tramadol				^a^				^a^		^a^ MI, PA	

*Key: For the prescription drugs*,

a is an identified substrate of the enzyme/protein;

b inhibits enzyme/protein activity;

c enhances enzyme/protein activity;

d *increases plasma levels of transaminases. The two letter codes denotes a herb which also affects the same PK target of the prescription drug: MI, Mangifera indica (Mango); VA, Vernonia amygdalina (Bitter leaf); OG, Ocimum gratissimum L. (Scent leaf); PN, Picralima nitida (Akuamma plant); AE, Abelmoschus esculentus (Okro); PA, Persea americana (Avocado pear); TC, Terminalia catappa (Tropical almond)*.

Concurrent use of *Albemoschus esculentus* (okro) with metformin is a case to highlight, given that the potential HDI is based on *in vivo* experimental evidence and because of the extensive use of the drug by diabetic patients. Although the experiment was carried out only with metformin, given that AE decreases intestinal absorption, it could also affect any other co-administered drug. *Persea americana* (Avocado) leaves, *Vernonia amygdalina* (bitter leaf), and *Mangifera indica* (mango) leaves interfere with the P-glycoprotein (P-gp) efflux pump and/or cytochromes 1A2 and 3A4 and could subsequently affect any drugs dependent on these enzymes and/or transporter. In addition, it can be seen from the table that mango leaves have the potential to interact with almost all the drugs in the table, since every drug is either metabolized by CYP 3A4 or is a substrate for P-gp. Finally, ingestion of *Ocimum gratissimum* (scent) leaf, *Picralima nitida* (akuamma) plant, and/or *Terminalia catappa* (tropical almond) could enhance the adverse hepatic effects of pioglitazone and atorvastatin.

With the above information, a practical assessment of potential HDI is herein carried out for one of the interviewed respondents “X,” a 41 year old lady who was taking the squeezed extract of *Vernonia amygdalina* (VA) regularly as well as an overnight decoction of *Mangifera indica* (MI) and had the following drugs on her prescription—Metformin 1 g twice daily, Glimepiride 4 mg daily, Lisinopril 2.5 mg daily, Atorvastatin 20 mg daily, and Aspirin 75 mg daily. A combination of the inhibitory effects of VA and MI on P-gp efflux activity as well as the ability of metformin to decrease P-gp expression (Kim et al., 2011), could bring about an overall increase in the plasma concentration of atorvastatin beyond the expected level, given that atorvastatin is a known P-gp substrate. Atorvastatin is however also dependent on CYP3A4 enzyme activity for metabolism to its active metabolite (Lennernäs, 2003). With significant CYP3A4 inhibition by MI, the clinical effect of atorvastatin would not be seen despite its high plasma concentration given that the metabolite is the pharmacologically active entity.

With this knowledge of the pharmacokinetics of her herbal medicines as highlighted in Table 3; and based on the information provided in Table 4, a recommendation to discontinue the use of MI would be provided for this patient in this case so as not to affect the therapeutic benefits of atorvastatin given that its metabolism is absolutely essential to its effect. On the other hand, there might not be a need to discontinue her use of VA unless its effect on P-gp is going to result in a significant increase in the plasma concentration of atorvastatin. For this, a dosage adjustment may then be required particularly where clear evidence of the medicinal plant has been shown.

## Discussion

The increased use of herbal medicines by patients alongside their prescription drugs has raised concerns for HDIs, which may be clinically relevant. By introducing a new and different approach to the results of our field study, we have carried out an investigation into the possible risks of pharmacokinetic HDIs amongst patients with diabetes in Nigeria; as well as a practical example with one patient of how such an assessment can be carried out given available information. One of the key findings of our study was that over 50% of diabetic patients in Nigeria use herbal medicines alongside their conventional drugs for their disease management, which highlights the large number of patients at risk of HDI and the need for such assessments. More worrying is that of this high number, more than 60% are unaware of the identity of the herbal medicines being taken which in turn highlights a complete ignorance of the risks of HDIs.

None of the observed variables—age, gender, cost of disease management, and presence of co-morbidities was identified as a predictor of herbal medicine use; and therefore could not be used as a means of easily identifying patients at risk of HDIs. Based on the study carried out by Nahin et al. (2012), we had expected an association between the number of co-morbidities (which directly relates to disease severity in the patient) and the use of herbal medicine. The absence of this, even though our study identified more users of herbal medicine amongst patients with two or more co-morbidities, could therefore attribute non-clinical factors as predictors of herb use.

Previous studies have indeed shown that the use of herbal medicines by an individual is very closely linked to his/her cultural health beliefs (van Andel and Westers, 2010). This is also directly related to one's perception about the health benefits of herbal medicines. A patient is therefore more likely to use herbal medicine if such practice is common in his/her family and if he/she is convinced of its efficacy. Thus, the sources of influence and people's personal opinion about herbal medicine could provide better prediction of herb use within a population. Given that these factors are not in themselves directly or clinically measurable, it further highlights the need for proactive approaches from healthcare practitioners to enhance increased communication with patients about their use of herbal medicines. One limitation to our study was the absence of information about non-clinical/cultural variables of the patients, such as educational background, demography, occupation and beliefs; as these would have enabled a better assessment of their influence on the use of herbal medicines.

An analysis of the drugs commonly prescribed to patients with diabetes show that most of these are substrates or modulators of known pharmacokinetic parameters commonly implicated in drug interactions. Studies have shown that the risk of harmful drug interactions increases with the number of drugs given to a patient (Fakeye et al., 2008). Similarly, the risk of HDIs is greatly increased for those patients taking herbal medicines who are on more than one prescription drug (Patsalos et al., 2002). The analysis done in Table 4 was only carried out for the drugs used by the patients for diabetes and diabetic-related co-morbidities. However, the complexities of potential drug interactions could increase further when one begins to consider medications for malaria or an antibiotic for an infection as well as multivitamins that diabetic patients may also be taking alongside.

We recently conducted an extensive review of medicinal plants used in diabetes management in Nigeria. One of the findings of the study was the paucity of information on investigations into pharmacokinetic interactions of commonly administered herbal medicines, compared to experiments validating its pharmacological activity (Ezuruike and Prieto, 2014). Given the high percentage of use, the complex composition of most herbal medicines and the chronic nature of the disease, there is a high risk of unidentified HDIs amongst patients with diabetes. This makes a case for the need for researchers to re-direct their focus into evaluating commonly used medicinal plants for both their pharmacodynamic and pharmacokinetic effects.

We have also shown how a true assessment of the risk of HDIs can be possible with the knowledge of a patient's herbal medicine. Healthcare practitioners who are aware of their patients' use of herbal medicines and its identity can more easily monitor them for any possible interactions using available information, such as that presented in Tables 3, 4. In addition to the expectation of an enhanced therapeutic effect for herbal medicines which are being taken for the same indication as the prescription drug (increased hypoglycaemia as in the case of diabetes); one should also be on the lookout for any associated pharmacokinetic interactions. Such information could result in possible dose adjustments to accommodate the added benefit of the herbal medicine, or a complete withdrawal if harmful. This is even more important for HDIs involving the inhibition or induction of cytochrome enzymes which are required for drug conversions to the more active metabolites or the elimination of pharmacologically active compounds. Clinically relevant drug interactions have been identified between the anti-hypertensive drug losartan and grapefruit juice (GFJ), because of the inhibitory effects of GFJ on CYP 3A4 and the dependence of the former on CYP3A4 for conversion to its active metabolite E3174 (Zaidenstein et al., 2001).

It is important to mention here that the availability of reliable data is required to adequately determine the HDI liability of herbal medicines. As with the case of GFJ and losartan above, this was validated by evidence from the clinical study. However, data obtained *in vitro* is very often required to plan and undertake a robust clinical assessment. In addition, it has now been shown that given the availability of *in vitro* data, physiologically based pharmacokinetic (PBPK) modeling and simulation tools can be used as an alternative means for the quantitative prediction of HDIs without the need for clinical studies (Brantley et al., 2014; Gufford et al., 2015).

Unfortunately, the high percentage of users of unidentified herbal medicines due to non-disclosure by herbalists makes the task of therapeutic monitoring of herbal medicines difficult, an issue that is unlikely to be immediately tackled. This is because traditional medicine practitioners/herbalists who prepare the herbal preparations themselves insist that it is a “family secret” of which a disclosure will rob them of their business (Ogbera et al., 2010). They are therefore often rightly reticent to reveal all the ingredients of their remedies, because of the history of “bio-piracy” by companies seeking to patent and profit from their knowledge without sharing benefits (Willcox and Bodeker, 2010). This non-disclosure is a limitation to the integration of traditional medicine in conventional healthcare systems. For this reason, patients should be advised on the importance of knowing the identity of their herbal medicines especially as this would enable a proper identification of those with true therapeutic benefits.

## Conclusion

Given the increasing prevalence of herbal medicine use alongside prescription medicines for disease management, this is the first time a study has been carried out specifically looking to identify potential HDIs amongst diabetic patients in Nigeria. We identified a high percentage (50%) of patients who use herbal medicines, and by so doing are exposed to a number of pharmacokinetic interactions. The lack of clinical predictors point toward non-measurable cultural factors influencing the use of herbal medicines by Nigerian adult diabetic patients. The identified pharmacokinetic HDIs are therefore highlighted here as a template for HDI assessment as well as to facilitate a more proactive monitoring by healthcare professionals.

## Author contributions

UE researched data and wrote the manuscript. JP contributed to the discussion and reviewed/edited the manuscript.

### Conflict of interest statement

The authors declare that the research was conducted in the absence of any commercial or financial relationships that could be construed as a potential conflict of interest.

## Abbreviations

ALT: Alanine transaminase
AST: Aspartate transaminase
CYP: Cytochrome P450
GSH: Glutathione
GST: Glutathione-S-transferases
HDI: Herb-drug interactions
P-gp: P-glycoprotein.
