# Supplementary material for: Assessment of Potential Herb-Drug Interactions among Nigerian Adults with Type-2 Diabetes
Source: Front Pharmacol. 2016 Aug 10;7:248. doi: 10.3389/fphar.2016.00248 (PMC4978708; doi:10.3389/fphar.2016.00248)
Supplement: Supplementary file 1 [file DataSheet1.DOCX]

### General information from respondents

- Name initials of Individual
- Sex of Individual
- Town of birth
- Town where they live
- Age of Individual (15-30, 30-45, 45-60, Above 60)
- Languages spoken
- Occupation (main and others)

### Proposed question(s) for diabetic patients

1. When did you start receiving treatment for diabetes?
2. What medications have been prescribed for you?
3. Are you also taking medications for any other ailment? If so, what are they?
4. Where do you obtain your medications and what are the cost implications to you every month for them?
5. Do you take any form of herbal preparations either for diabetes or any other ailment alongside your prescription drugs? If so, what are they and how often do you take them?
6. How do you obtain your herbal preparations and how much do you pay for them?
7. Do you sometimes skip doses of any of the above mentioned means of treatment? If so, why?
8. What treatment do you consider more effective (or do you prefer)?
9. Have you had any side effects to any of your prescription medicines, either currently or in the past?
10. Have you had any side effects to any herbal preparations, either currently or in the past?

**I HEREBY CONSENT TO THE ABOVE INFORMATION BEING USED FOR RESEARCH PURPOSES.**

**________________________**

**SIGNATURE**
